# Cognitive Capacity, Representation, and Instruction

**DOI:** 10.3389/fpsyg.2021.701687

**Published:** 2021-07-20

**Authors:** Thomas Kleinsorge

**Affiliations:** Department Psychology and Neurosciences, Leibniz Research Centre for Working Environment and Human Factors at the TU Dortmund (IfADo), Dortmund, Germany

**Keywords:** cognitive capacity, representation, instruction, task space, cognitive resource

## Abstract

The central argument of the present article is that Cognitive Psychology’s problems in dealing with the concept of “cognitive capacity” is intimately linked with Cognitive Psychology’s long-lasting failure of coming to terms with the concept of “representation” in general, and “task representation” in particular. From this perspective, the role of instructions in psychological experiments is emphasised. It is argued that both a careful conceptual analysis of instruction-induced task representations as well as an experimental variation of instructions promises to broaden our understanding of the role of task representations as a determinant of limited cognitive capacity.

## Introduction

The central argument of this article is that the concept of “cognitive capacity” suffers from Cognitive Psychology’s long-lasting problems of coming to terms with the concept of “representation” in general, and “task representation” in particular. The notion of cognitive capacity refers to limits in cognitive processing and task performance that are thought to arise from limits intrinsic to an organism, with these limits being subject to intra- and interindividual variation.

In what follows, I will take the so-called “imagery debate” as a point of departure to discuss some fundamental problems of the concept of “representation.” Then, I will discuss these problems with respect to the way instructions in psychological experiments may work. This will lead me to the distinction between the extension and the intension of a (task) representation, which I link to my own previous work regarding the concept of “task space.” Ultimately, I will propose that the constraints inherent to task spaces may offer a representational account of some of the cognitive limitations that are usually discussed in terms of cognitive capacity. Due to its basically representational nature, this account sees limits of capacity not as a feature of an organism but as arising from organism-environment interactions as shaped by task representations.

### Problems of the Concept of “Representation”

In the 70s and 80s of the last century, there had been rigorous debates around the concept of “representation” in Cognitive Psychology. One point of culmination was the “imagery debate” which centred around questions of representational format, i.e., whether or inasmuch cognitive representations are implemented in a propositional, symbolic format akin to language or should be conceived as a direct, analogue mapping of properties of the environment on brain states (cf. Pylyshyn, 2002). In hindsight, it seems that the analogue-mapping account won the palm, but this could have been a Pyrrhic victory as many of the fundamental problems remained unresolved (cf. Slezak, 2002). At the same time, the emergence of connectionist modelling promised to provide a solution to the problem of representation by transferring it to a “sub-symbolic” level (cf. Smolensky, 1988).

In many parts of Cognitive Psychology, these developments resulted in “models” of cognitive processes that are based on codes for environmental properties (without caring much about where these come from) that are interconnected by excitatory and inhibitory connections [which are based more on knowledge about the outer world than on knowledge about the brain; cf. the commentaries to the target articles of Smolensky (1988) and Pylyshyn (2002)]. What is largely neglected, however, are issues of representational format. Are there intrinsic limits to what can be represented within a single coherent representation that are not merely reflections of the incongruity of certain environmental states? And if there are such limits of “representational capacity,” may they -at least in part- converge on limits of “cognitive capacity”?

From the perspective of instructable artificial systems, the distinction between symbolic and subsymbolic-connectionist systems has far-reaching consequences (cf. Noelle and Cottrell, 1995): While with symbolic systems “learning by being told” comes almost for free and boils down to a matter of translation between symbolic notations, this kind of rapid learning is hard to implement in a connectionist network due to its slow learning dynamics in terms of weight adaptation. This is not to say that it is impossible to implement such rapid learning into a (localist) connectionistic architecture, but this is usually done by assigning individual stimuli and responses to single units (cf. Ramamoorthy and Verguts, 2012). Thus, the mapping problem (see below) normally to be solved by the participant is solved by the designer of the connectionist architecture. On the other hand, within such an architecture inductive learning comes almost for free, which has to be formally implemented in symbolic architectures.

How are participants in psychological experiments disposed to (hopefully) implement those processes we aim to study? It is by instructions that are usually delivered in a verbal format. In some still largely mysterious way (most) participants are able to transform this verbal information into a format that allows them to perform the instructed task. Does this transformation preserve some of the structure of the verbal format of the original instruction? (To differentiate between verbal/symbolic and non- or sub-symbolic codes (more precisely, tokens of codes), the former but usually not the latter can be assumed to be endowed with some form of syntactical structure (i.e., not every token can enter into any relation with every other token) as well as compositionality (tokens with the same syntactical role are interchangeable in yielding legal expressions irrespective of whether the expressions refer to anything that exists).

### Representation and Instruction

In one of my earliest studies (Kleinsorge, 1999), I investigated the “orthogonal compatibility effect” (cf. Cho and Proctor, 2003) by varying the format in which the stimulus-response mapping of the respective upcoming trial was instructed. (The general instruction at the beginning of the experiment was given verbally.) The (visual) mapping instruction was either presented verbally or by a segment of a circle connecting stimulus and response positions. It turned out that the orthogonal compatibility effect was only observed with verbal but not with pictorial instructions. In a subsequent experiment, it could be shown that it was not the format of the instruction *per se* but the way participants processed this information: when participants received only instructions regarding the response assigned to one of the stimulus locations but had to generate the complementary stimulus-response mapping, the compatibility effect showed up again. Nevertheless, participants responded much faster with pictorial as compared to verbal instructions even with incomplete information, ruling out that the missing information was inserted in a verbal format. These observations suggest that a sequential processing of information, which is intrinsic to verbal information but had to be imposed with pictorial information, was critical for the emergence of the orthogonal compatibility effect.

These findings point to the importance of representational format for the efficiency of performing a certain task by demonstrating that essentially the same task can be represented in different formats that result in different levels of performance including the presence vs. absence of a specific compatibility effect (which is often considered as a limitation of the capacity to inhibit irrelevant information)However, in most cases we have no control of the format in which participants represent an instructed task, which also implies limited control of the way task-relevant information is processed inasmuch this processing is determined by the format in which this information is coded.

What we can take for granted is that the build-up of a task representation by participants usually starts with a verbal instruction, but we know little about the format of the resulting processing structure by which participants perform the instructed task. One possibility would be that participants simply “copy” the critical parts of the instruction (e.g., individual stimulus-response mappings) and verbally rehearse these in the course of the experiment (Goschke, 2000). When the critical parts of the instruction consist of rules (e.g., “press the right key if the stimulus is a word and the left key if it is a pseudoword”), these may be encoded and rehearsed in verbal working memory. There is evidence that when instructed either by individual stimulus-response mappings or rules, participants stick to the original way they have been instructed (cf. Dreisbach and Haider, 2009). This observation would be in line with the “copying account” sketched before, which can be considered as the simplest form of “learning by being told.” However, it is highly unlikely that such an account would be able to explain behaviour beyond the performance of simple lab tasks. Furthermore, recent evidence suggests that instructions that were initially stored in verbal working memory become rapidly stored in procedural memory by demonstrating that factors known to affect verbal working memory (phonological similarity, serial position) lose their impact after only a few trials of practice (Monsell and Graham, 2021).

When we assume that participants usually transform the verbal information of an instruction into some kind of internal format, two possibilities arise. Either, there is one -and only one- internal format enabling the formation of an effective task representation. This would mean that any situation directly determines its corresponding representation. This position would ultimately amount to a direct-coding account that comes along without any need for recoding the initially verbal information provided by the instruction. (This is not to say that the resulting task representation is verbal, but only that the representation formed on the basis of this information is solely dependent on situational affordances.) In this case, there would be no reason to worry much about instructions^1^ : learning by instruction boils down to a straightforward mapping problem that requires from the system (the participant) to find out which input should be mapped onto the activation of a certain output pattern (cf. Noelle and Cottrell, 1995). Importantly, this view shifts the process of implementing an instruction into a black box without any behavioural correlate (perhaps apart from some erratic behaviour in the very first trials of an experiment). However, it is fully obvious that we constrain this process by “telling” our participants. Ignoring this corresponds to ignoring the problem of commensurability of symbolic and sub-symbolic codes, an ignorance that, as outlined above, accompanies Cognitive Psychology for decades.

On the other hand, if it is assumed that information conveyed by instruction can be represented in different formats, the question arises whether different formats result in differently efficient task performance, and why this is the case. From dual-task research it is known that participants’ performance critically depends on whether the nominally two tasks allow to be represented as a higher-order single task (cf. Schmidtke and Heuer, 1997). If so, one may ask what it is that allows for the formation of such a higher-order task representation. At this point, it may be useful to refer to the distinction between the *extension* and the *intension* of a representation. This distinction goes back to Arnauld (1685/1972) and was applied to the problem of mental representations by Lundh (1981, 1982, 1995). The term intension refers to the relation of a mental (or neural) token to other tokens, or the relation of a concept to other concepts. Importantly, as such, intension lacks referential semantics, it is only about “connections” akin to connectionist networks. Referential semantics are provided by the extension of a representation, which is based on instantiations in perceptual and behavioural terms that link intensions to external referents. (On a neurophysiological level, intension seem to be represented primarily in the hippocampus (e.g., O’Reilly and Rudy, 2001)).

Interestingly, Lundh (1995) also proposed a solution to the above-mentioned imagery debate by assuming that intensions are stored in a unitary (one could also say: sub-symbolic) format, whereas extension is instantiated in different modality-specific codes. The latter assumption converges upon “embodied” accounts of cognition (e.g., Rosch et al., 1991; Wilson, 2002) that assume that cognitive processes are grounded in mechanisms of sensory processing and motor control that evolved for interaction with the environment (the extensional referential semantics in Lundh’s terms). However, whereas embodiment accounts provide a quite successful solution to the problem how internal codes are grounded in organism-environment interactions, they tend to neglect the problem of syntactical structure of intension.

### Task Representation and Cognitive Capacity

On these grounds, I suggest that the formation of a higher-order task representation critically depends on whether the lower-level tasks can concurrently be mapped on the same intensional configuration. This configuration is not to be confused with the much narrower concept of “task set” but corresponds more closely what Herbert Heuer and I (Kleinsorge and Heuer, 1999) termed “task space” (cf. Xiong and Proctor, 2018, for a thorough treatment of the distinction between task set and task space). Thus, metaphorically speaking, efficient performance of a complex task is dependent on being located in the same task space. However, as cogently explicated by Xiong and Proctor (2018), being located in the same task space also provides a basis for interference as the presentation of a stimulus may not only activate those aspects of a stimulus that are via instruction task relevant (as part of the task set) but activation may spread (via intensional relations) to task-irrelevant aspects that are thereby part of the task space. “Conflict tasks” of i.e. the Stroop- or Eriksen-type are specifically designed to induce interference which is then interpreted as indicating limits of cognitive capacity.

Beyond this conceptual level, the architecture of a certain task space may go along with certain ways of navigating it. One of our basic observations regarding a certain type of task combination (resulting from a factorial combination of two binary task dimensions) consisted of a certain pattern of costs for switching among the subtasks of this task space (cf. Kleinsorge et al., 2004). We accounted for this pattern by assuming a certain “hierarchical switching mechanism” that results in instantaneous “co-switches” when a higher-level task feature switched (cf. Korb et al., 2017, for recent neurophysiological evidence supporting the existence of such a mechanism).

To add another example from my own research: There is some quite compelling evidence that task switching proceeds much more efficiently when the next task is indicated by an explicit task cue as compared to mere foreknowledge of the task sequence (e.g., Koch, 2003). This comes along as a classical “capacity limitation” with respect to advance preparation. However, we have shown that this “capacity limitation” is restricted to switching among only two tasks. When switching among four tasks, this difference disappears, probably due to a richer “intensional” representation of the differences among four (as compared to two) tasks (cf. Kleinsorge and Apitzsch, 2012). Thus, what may be considered as a capacity limitation (in terms of endogenous preparation) may be due to a mismatch of (experimenter-presented) external stimulation and internal processing structure (in case of memory-based, task switching among two tasks).

## Conclusion

If it is true that in psychological experiments all begins with instructions which then are to be transformed into an internal representation, it seems obvious that instructions strongly determine the general lay-out of a task space (cf. Xiong and Proctor, 2018). Given this, it seems to be surprising that we as experimental psychologists pay so little attention to instruction, either by conceptual analyses as outlined above, or by way of varying (parts of) instruction in a systematic manner (e.g., Hommel, 1993; Kleinsorge, 1999, 2009; Dreisbach and Haider, 2009)^2^.

In some way, we as experimental cognitive psychologists, are funny creatures: We lead our participants to perform awfully simple “tasks” to investigate the limits of “cognitive capacity,” while at the same time we and the people around us routinely perform highly complex actions in the pursue of even more complex task goals—and it seems that we do not even wonder.

## Data Availability Statement

The original contributions presented in the study are included in the article/supplementary material, further inquiries can be directed to the corresponding author/s.

## Author Contributions

TK contributed to this article.

## Conflict of Interest

The author declares that the research was conducted in the absence of any commercial or financial relationships that could be construed as a potential conflict of interest.

## Footnotes

1 Apart from representational issues, instructions are also a social agent providing researchers and their participants with common ground for their interaction (cf. Gozli, 2019).

2 This is not to say that there is no relevant work on the effects of instructions. Much of this work centres around questions inasmuch representations of instructions are stored in verbal working memory (e.g., Monsell and Graham, 2021), or inasmuch interference exerted by a competing instructed task depends on executing this task as compared to being instructed to execute the task in the future (e.g., Liefooghe et al., 2012). I consider these approaches as complementary to the one presented here.

## References

[B1] Arnauld A. (1685/1972). *Die Logik oder Die Kunst des Denkens [Logic or the Art of Thinking] (frz. Orig. 1685).* Darmstadt: Wissenschaftliche Buchgesellschaft.

[B2] Cho Y. S. Proctor R. W. (2003). Stimulus and response representations underlying orthogonal stimulus-response compatibility effects. *Psychon. Bull. Rev.* 10 45–73. 10.3758/bf03196468 12747491

[B3] Dreisbach G. Haider H. (2009). How task representations guide attention: further evidence for the shielding function of task sets. *J. Exp. Psychol.* 35 477–486. 10.1037/a0014647 19271860

[B4] Goschke T. (2000). “Involuntary persistence and intentional reconfiguration in task-set switching,” in *Attention and Performance XVIII: Control of Cognitive Processes*, eds Monsell S. Driver J. (Cambridge, MA: MIT Press), 331–355.

[B5] Gozli D. (2019). *Experimental Psychology and Human Agency.* Cham: Springer.

[B6] Hommel B. (1993). Inverting the Simon effect by intention. *Psychol. Res.* 55 270–279. 10.1007/bf00419687 8416040

[B7] Kleinsorge T. (1999). Die Kodierungsabhängigkeit orthogonaler Reiz-Reaktions-Kompatibilität [Coding specificity of orthogonal S–R compatibility]. *Z. Exp. Psychol.* 46 249–264. 10.1026//0949-3964.46.4.249 10551040

[B8] Kleinsorge T. (2009). Switching among two-component tasks. *Adv. Psychol. Res.* 59 1157–1174.

[B9] Kleinsorge T. Apitzsch N. (2012). Task preparation based on precues versus memory: precues lead to superior performance with two tasks but not with four tasks. *J. Cogn. Psychol.* 24 140–156. 10.1080/20445911.2011.598855

[B10] Kleinsorge T. Heuer H. (1999). Hierarchical switching in a multi-dimensional task space. *Psychol. Res.* 62 300–312. 10.1007/s004260050060

[B11] Kleinsorge T. Heuer H. Schmidtke V. (2004). Assembling a task space: global determination of local shift costs. *Psychol. Res.* 68 31–40. 10.1007/s00426-003-0134-9 12774231

[B12] Koch I. (2003). The role of external cues for endogenous advance reconfiguration in task switching. *Psychon. Bull. Rev.* 10 488–492. 10.3758/bf03196511 12921429

[B13] Korb F. M. Jiang J. King J. A. Egner T. (2017). Hierarchically organized medial frontal cortex-basal ganglia loops selectively control task-and response-selection. *J. Neurosci.* 37 7893–7905. 10.1523/jneurosci.3289-16.2017 28716966 PMC5559764

[B14] Liefooghe B. Wenke D. De Houwer J. (2012). Instruction-based task-rule congruency effects. *J. Exp. Psychol.* 38 1325–1335. 10.1037/a0028148 22545614

[B15] Lundh L. G. (1981). The mind considered as a system of meaning structures: elementary meaning structures. *Scand. J. Psychol.* 22 145–160. 10.1111/j.1467-9450.1981.tb00389.x 7156910

[B16] Lundh L. G. (1982). The mind considered as a system of meaning structures II. Concepts and representations. *Scand. J. Psychol.* 23 225–242. 10.1111/j.1467-9450.1982.tb00436.x 7156910

[B17] Lundh L. G. (1995). Meaning structures and mental representations. *Scand. J. Psychol.* 36 363–385. 10.1111/j.1467-9450.1995.tb00994.x 7156910

[B18] Monsell S. Graham B. (2021). Role of verbal working memory in rapid procedural acquisition of a choice response task. *Cognition* 214:104731. 10.1016/j.cognition.2021.104731 33992845

[B19] Noelle D. C. Cottrell G. W. (1995). “Towards instructable connectionist systems,” in *Computational Architectures Integrating Neural and Symbolic Processes*, eds Sun R. Bookman L. A. (Boston, MA: Springer), 187–221. 10.1007/978-0-585-29599-2_6

[B20] O’Reilly R. C. Rudy J. W. (2001). Conjunctive representations in learning and memory: principles of cortical and hippocampal function. *Psychol. Rev.* 108 311–345. 10.1037/0033-295x.108.2.311 11381832

[B21] Pylyshyn Z. W. (2002). Mental imagery: in search of a theory. *Behav. Brain Sci.* 25 157–238. 10.1017/s0140525x02000043 12744144

[B22] Ramamoorthy A. Verguts T. (2012). Word and deed: a computational model of instruction following. *Brain Res.* 1439 54–65. 10.1016/j.brainres.2011.12.025 22264490

[B23] Rosch E. Varela F. Thompson E. (1991). The embodied mind. Cognitive science and human experience.

[B24] Schmidtke V. Heuer H. (1997). Task integration as a factor in secondary-task effects on sequence learning. *Psychol. Res.*, 60 53–71. 10.1007/BF01792433 8810586

[B25] Slezak P. (2002). The tripartite model of representation. *Philos. Psychol.* 15 239–270. 10.1080/0951508021000006085

[B26] Smolensky P. (1988). On the proper treatment of connectionism. *Behav. Brain Sci.* 11 1–23. 10.1017/s0140525x00052432

[B27] Wilson M. (2002). Six views of embodied cognition. *Psychon. Bull. Rev.*, 9 625–636. 12613670 10.3758/bf03196322

[B28] Xiong A. Proctor R. W. (2018). “The role of task space in action control: evidence from research on instructions,” in *Psychology of Learning and Motivation*, Vol. 69 ed. Federmeier K. D. (San Diego, CA: Academic Press), 325–364. 10.1016/bs.plm.2018.09.007

